# Brain raspberries: a microvascular finding, seen with anti-collagen 4 staining

**DOI:** 10.1016/j.cccb.2026.100559

**Published:** 2026-08-19

**Authors:** Hannah Storbråten Edstam, Henric Ek Olofsson, Elisabet Englund

**Affiliations:** Division of Pathology, Department of Clinical Sciences Lund, Lund University, Sölvegatan 25B, 22185, Lund, Sweden

**Keywords:** Cerebrovascular disease, Aging, Angiogenesis, Microvascular proliferation

## Abstract

**Introduction:**

A brain raspberry is a microvascular formation of yet insufficiently known origin and significance, defined histologically as three or more transversally sectioned vascular lumen surrounded by a common perivascular space. Previous reports on the prevalence of this structure have been based mainly on histopathological routine staining with haematoxylin-eosin (HE). The aim of this study was to compare the detectability of raspberries between HE and immunohistochemical vessel staining with anti-collagen 4 (col4).

**Materials and methods:**

We used 50 pairs of tissue sections from the cerebral cortex of 40 deceased individuals who had undergone a diagnostic neuropathological examination at the Department of Pathology, Lund, Sweden, during 2019–2022. Raspberries were manually quantified on digitally scanned cortical sections stained with HE and col4, respectively.

**Results:**

Staining with col4 showed a higher detectability of cortical raspberries than staining with HE, with an absolute difference of 12.1 raspberries/cm^2^ and a relative difference of 252.1%. The difference between the two staining methods was statistically significant (P < 0.001).

**Conclusions:**

This study reveals that staining with col4 provides a substantially higher detectability of raspberries compared with HE staining. Immunohistochemical detection is recommended for future studies on the pathogenesis and prevalence of brain raspberries.

## Highlights

• Microvascular brain raspberries are of potential pathological significance.
• Existing literature is based mainly on haematoxylin-eosin staining.
• Staining with collagen 4 provided significantly higher detectability of brain raspberries.

## 1 Introduction

During recent years, a relatively unknown histopathological alteration called microvascular proliferation or “raspberry” has been regularly observed in the brain, mainly the cerebral cortex, during neuropathological investigations at the Department of Clinical Pathology in Lund. The name ‘raspberry’ originates from its berry-like appearance under a bright-field microscope. Raspberries have been defined as a structure with a minimum of three transversally sectioned adjacent microvascular lumen, surrounded by a common perivascular space [[1], [2], [3], [4]]. It should be noted that this finding has been referred to by different terms in the literature, including the recent ‘multi-lumen vascular profiles’ [5]. ‘Raspberry’ will be used throughout this text to remain consistent with the terminology from our earlier publications on this topic [[1], [2], [3], [4],^6^].

In previous studies, it has been shown that an increasing cortical density of raspberries correlates with advancing age [4,5,[7], [8], [9]]. Our group also reported a positive correlation between raspberries and cerebral atherosclerosis of the basal cerebral vessels (including the circle of Willis), also with vascular dementia and frontotemporal lobar degeneration (FTLD) [[1], [2], [3]]. Raspberries may, in our experience, constitute up to 3% of the cortical blood vessels [6].

**Fig. 1:**
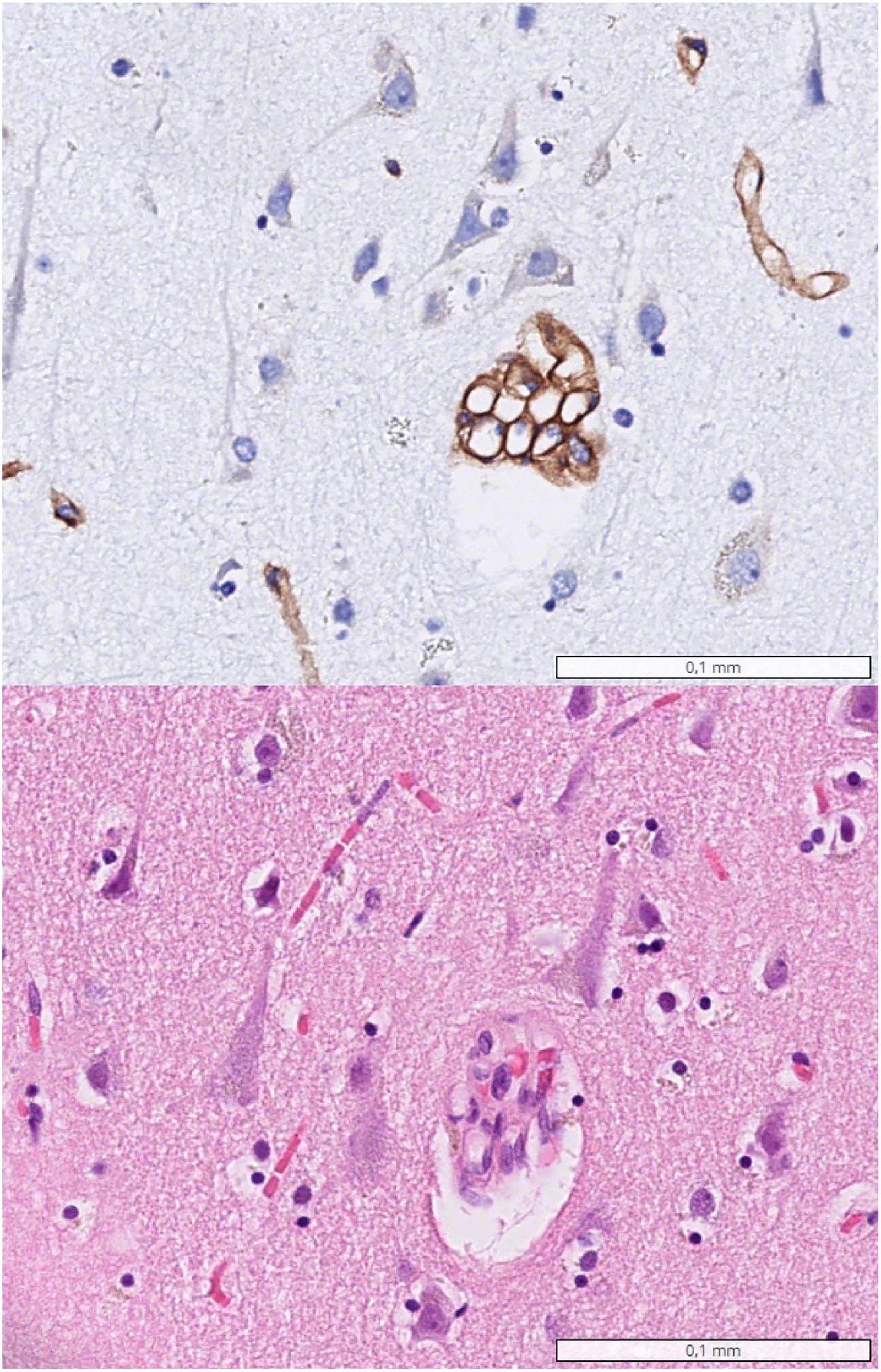
Low power microphoto of a raspberry stained immunohistochemically for collagen 4 (upper) and a raspberry stained with standard Haematoxylin-eosin (lower).

Raspberries can be detected with standard haematoxylin-eosin (HE) staining but in our experience, they may be even more readily seen with immunohistochemical labelling of collagen 4 (col4) – the most abundant protein in the vascular basement membrane [1,10] (Fig. 1).

The aim of this study was to explore the potential advantage of immunohistochemical col4-detection over HE for quantifying raspberries by performing a direct comparison between these two staining methods.

## 2 Materials and methods

### 2.1 Study population

We examined 50 pairs of brain cortical sections (frontal and occipital) from 40 deceased adult individuals who had undergone a clinical autopsy including a neuropathological examination at the Department of Pathology in Lund, Sweden between 2019 and 2022. In these 40 individuals, a finding of raspberries (any quantity) had been described in the autopsy report, hence these were chosen for closer analysis and quantification. Each pair of sections was stained with HE and col4 (Agilent/Dako; CIV 22; M0785), yielding a total of 100 stained sections for comparison.

The study population consisted of 26 male and 14 female individuals, with a median age at death of 74 years (ranging between the ages of 69–80). Twenty of these cases had a neuropathological diagnosis of FTLD, including both tau and non-tau pathologies [11]. The remaining 20 individuals were cognitively unimpaired according to the autopsy referral text and to other available clinical data in medical records. The neuropathological findings observed in this group were heterogeneous and included infectious, hypoxic-ischaemic and neoplastic lesions, as well as cases where no significant pathological findings were identified.

### 2.2 Raspberry quantification

**Fig. 2:**
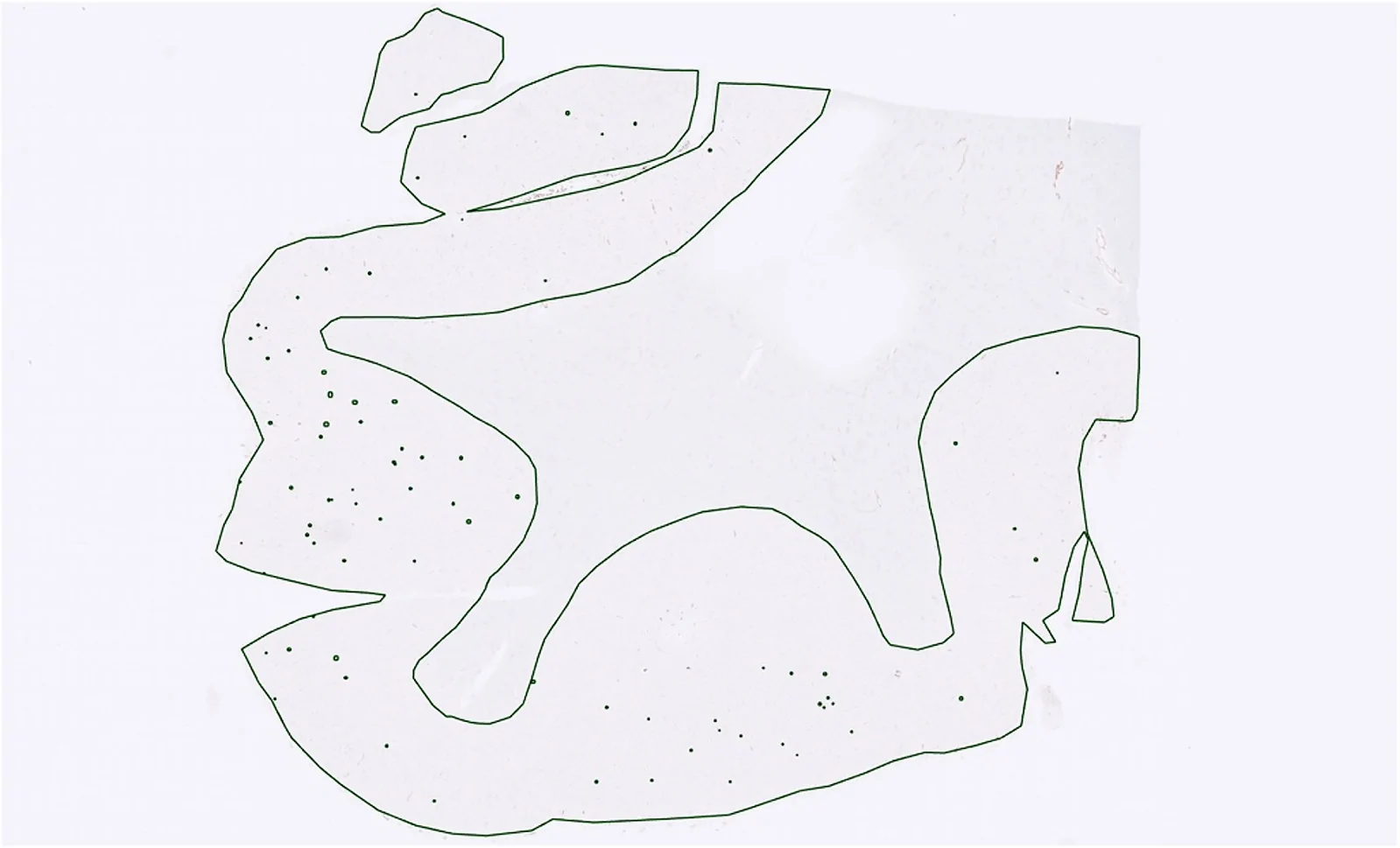
Overview of a tissue section from the cerebral cortex, stained immunohistochemically with an antibody against collagen 4. The cortex area has been delineated, and detected raspberries have been marked.

Whole slide images of scanned tissue sections were examined digitally in the web-based image data system Sectra IDS7. The area of the cerebral cortex of each section was measured and the raspberries within this area were marked and quantified (Fig. 2*).* To be included as a raspberry, a structure had to fulfil the previously stated definition of a raspberry, i.e. a minimum of three transversely sectioned adjacent microvascular lumina surrounded by a common perivascular space. The average area examined on the sections was 148mm^2^. The number of raspberries found was correlated with the area of the examined cortex and transformed to values with the unit raspberries/cm^2^.

### 2.3 Statistics

Statistical analyses were made with IBM SPSS Statistics Version 29. Non-parametric testing with Wilcoxon Signed Rank test was used due to skewed data.

A p-value of < 0.05 was considered statistically significant.

### 2.4 Ethical approval

Approval was given by the Swedish Ethical Review Authority for the entire raspberry project, of which this project was a sub-study. The main application was accepted in 2019, followed by two amendments in 2021 and 2023 (application number 2021-05753-02 and 2023-03097-02).

## 3 Results

In all, quantitation was made on sections from 50 paraffin blocks where two stainings were compared (i.e. 100 sections). The brain tissue represented individuals in which at least one brain raspberry had been reported in the clinical autopsy investigation.

**Fig. 3:**
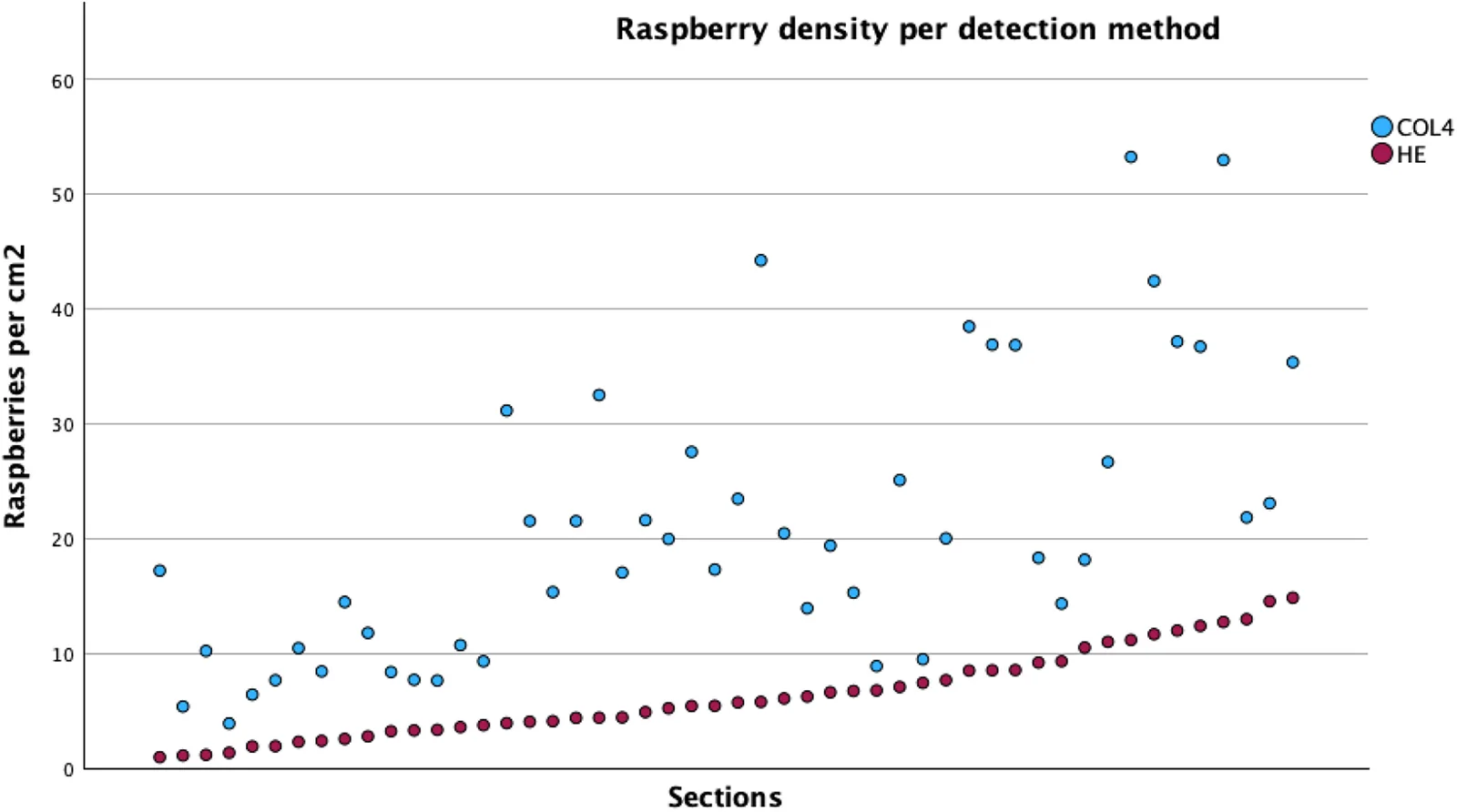
Cortical raspberry density of individual sections according to staining with col4 (blue) and HE (red). Each x value contains the data from one study participant. col4 = collagen 4; HE = Haematoxylin-eosin.

We found a statistically significant, higher detectability with the col4 staining than with the HE staining (p < 0.001). The median raspberry density in the sections stained with HE was 5.6 raspberries/cm^2^ (ranging between 1.0 – 14.9) whilst in sections stained with col4 the median raspberry density was 18.9 (ranging between 3.9 – 53.2). Hence staining with col4 showed a higher detectability, with a median absolute difference of 12.1 raspberries/cm^2^ (ranging between 2.1 – 42.0) and a median relative difference of 252.1%. (Table 1, Fig. 3)

**Table 1:** Raspberry density in 50 paired sections stained with anti-col4 and HE.

**Raspberry density**		
	**COL4-staining** (raspberries/cm^2^)	**HE-staining** (raspberries/cm^2^)
**Median** (min-max)	**18.9** (3.9 – 53.2)	**5.6** (1.0–14.9)
**Absolute difference** (min-max)	**12.1** (2.1 - 42.0)	
**Relative difference** (%)	**252.1%**	

col4 = collagen 4; HE = Haematoxylin-eosin.

## 4 Discussion

Immunohistochemical staining with col4 labels the vascular basement membrane, and the here described raspberries represent a vascular formation of a type not yet fully mapped or known. While HE presents almost all tissue components, among which the raspberries can be found, this staining does not specifically target blood vessels. The higher detectability of raspberries with col4 staining was somewhat foreseen [1,5], yet it was not previously shown to be statistically significant.

It should possibly be of pathogenetic interest to analyse the variability of immunohistochemical staining intensity and to compare different components of the basement membrane [12] as well as the presence of smooth muscle actin, which would add to the collagen 4 in the maturation of an arteriolar vessel wall, at contrast with a newly proliferated vessel. If also capillaries are found to be part of the microvascular proliferative change (which is suspected, however not yet fully established), the same possibility to date the vascular alteration would not apply due to the different vascular wall structures.

## 5 Conclusion

Based on a study population of 40 adult individuals with varying neuropathological findings, this study concludes that staining with col4 provides a substantially higher detectability of raspberries compared with HE and hence col4 should be the staining to prefer in future studies that include raspberry quantification. The pathobiology of these alterations deserves further studies. It is motivated to analyse immunohistochemical staining intensity and compare basement membrane components in an attempt to distinguish old from newly formed vessels.

## Funding

This study was funded by the Trolle-Wachtmeisters’s Foundation for Medical Research (EE), grant 2024-25.

## CRediT authorship contribution statement

**Hannah Storbråten Edstam:** Writing – original draft, Visualization, Methodology, Investigation, Formal analysis, Data curation. **Henric Ek Olofsson:** Writing – review & editing, Validation, Methodology, Investigation, Conceptualization. **Elisabet Englund:** Writing – review & editing, Validation, Supervision, Software, Resources, Project administration, Investigation, Funding acquisition, Data curation, Conceptualization.

## Declaration of competing interest

Regarding our submission on the article “Brain raspberries: a microvascular finding, seen with anti-collagen 4 staining” we don’t have any specific interests to make statements about.
